# Strong Association of Waist Circumference (WC), Body Mass Index (BMI), Waist-to-Height Ratio (WHtR), and Waist-to-Hip Ratio (WHR) with Diabetes: A Population-Based Cross-Sectional Study in Jilin Province, China

**DOI:** 10.1155/2021/8812431

**Published:** 2021-05-14

**Authors:** Fu-Liang Zhang, Jia-Xin Ren, Peng Zhang, Hang Jin, Yang Qu, Yao Yu, Zhen-Ni Guo, Yi Yang

**Affiliations:** ^1^China National Comprehensive Stroke Center, Department of Neurology, The First Hospital of Jilin University, Xinmin Street No. 1, Changchun 130021, China; ^2^Clinical Trial and Research Center for Stroke, Department of Neurology, The First Hospital of Jilin University, Xinmin Street No. 1, Changchun 130021, China; ^3^Jilin Provincial Key Laboratory, The First Hospital of Jilin University, Xinmin Street No. 1, Changchun 130021, China

## Abstract

**Backgrounds:**

The prevalence of diabetes has increased with the increase of obesity, and finding indicators to predict diabetes risk has become an urgent need. The purpose of this study is to compare the correlation between four anthropometric indices and the prevalence of diabetes.

**Methods:**

A total of 4052 participants aged 40 years and above were selected in Dehui City, Jilin Province, using a multistage stratified whole group sampling method. Face-to-face interviews and physical examinations were conducted. Multivariate logistic analysis was used. The values of BMI, waist circumference (WC), waist-to-hip ratio (WHR), and waist-to-height ratio (WHtR) were divided into quartiles (Q1: <25%; Q2: ~25%; Q3: ~50%; and Q4: ~75%). The median of each quartile was used for a linear trend test.

**Results:**

For all four body fat-measuring indices of body mass index (adjusted OR: 3.300, 95% CI: 2.370, 4.595), WC (adjusted OR: 5.131, 95% CI: 3.433, 7.669), WHR (adjusted OR: 3.327, 95% CI: 2.386, 4.638), and WHtR (adjusted OR: 5.959, 95% CI: 3.922, 9.054), patients in the highest quartile were more likely to have diabetes than those in the lowest quartile. The areas under the curve of WHtR, WC, WHR, and BMI for diabetes were 0.683, 0.669, 0.654, and 0.629, respectively. In female participants, the areas under the curve of the waist-height ratio and WC were 0.710 (95% CI: 0.679-0.741) and 0.701 (95% CI: 0.670-0.732), respectively.

**Conclusions:**

The WC and WHtR were more closely related to diabetes than BMI and WHR among study participants ≥ 40 years of age, especially in females.

## 1. Introduction

In the past decade, diabetes has become a major public health burden worldwide [1]. Due to an aging population, urbanization, nutritional changes, decline in physical activity levels, and the prevalence of obesity along with a large population, the burden of diabetes in China is rapidly increasing, especially type II diabetes (90-95% of diabetics) [2, 3]. Epidemiological studies [4] have shown that in 2010, the number of Chinese adults with diabetes reached 43.2 million [5]. It is predicted that by 2030, China will have 62.6 million people with diabetes. Since diabetes increases the risk of microvascular and macrovascular complications and premature death in the general population, there is an urgent need for accurate assessment of methods for diabetes prevention, early detection, and diagnosis [6]. Risk factors for diabetes that largely contribute to its occurrence are age, family history of diabetes, high blood lipid levels, obesity, dietary habits, and insufficient physical exercise [7], among which obesity is an important risk factor for diabetes [8]. Abdominal obesity is closely related to insulin resistance and diabetes [9]. Excessive ectopic fat distribution in sites such as the viscera leads to abnormal fat metabolism and aggravates the development of diabetes [10]. Clinical evidence has revealed that in the Chinese population, the association of central obesity with diabetes is stronger than that of ordinary obesity [11]. Therefore, screening for diabetes by utilizing anthropometric indicators and parameters that reflect abdominal fat has become a focus of research.

Generally, the status of body fat accumulation can be described by four indicators: waist circumference (WC), body mass index (BMI), waist-to-height ratio (WHtR), and waist-to-hip ratio (WHR). BMI is calculated as weight/height^2^ [12]. WHtR is the ratio of waist circumference to height, and WHR is the ratio of waist circumference to hip circumference. Research studies have reported that the prediction of each anthropometric index depends on the population and varies with different races [13, 14]. In China, the population and economic development levels of different provinces are quite different, and therefore, there is a big variation in the prevalence of diabetes. Data in 2013 showed that compared to other provinces, the disease burden caused by high blood sugar in Jilin Province was more serious (population attributable fraction [PAF] = 9.94) [15, 16]. However, there are few studies comparing the values of these four anthropometric indices and their relationship with the prevalence of diabetes. Consequently, the relationship between the incidence of diabetes and obesity indicators in Jilin Province is unclear. It is also worth noting that there is no consensus on the value of many human body indicators for predicting diabetes, and it is still controversial [17, 18]. For different groups of people, these anthropometric indices for predicting diabetes play different roles. Some studies believe that WHtR can be used as the best indicator for undiagnosed type 2 diabetes [19]. In contrast, Yang et al. have shown that BMI is the strongest predictor of diabetes in men and women [20], and Wang et al. have indicated that WC is more effective than WHR for predicting diabetes [21]. Therefore, comparing the relationship between diabetes and the four anthropometric indicators is important to guide the preliminary prevention and early detection of diabetes in Jilin Province. This study was aimed at comparing the relationship between the four anthropometric indicators and diabetes in areas with a high incidence of diabetes in Jilin Province, along with the effectiveness of the different indicators in diagnosing diabetes, by inferring the optimum cut-off values and differences due to sex of the participants.

## 2. Materials and Methods

### 2.1. Study Design and Population

The study population was derived from the Stroke Screening and Prevention Program of the National Health and Family Planning Commission of China, which was one of the National Key Technology Research and Development programs (grant No. 2011BAI08B01) supervised by the Chinese National Centre for Stroke Care Quality Control and Management [22, 23]. The detailed program has been published before [24]. This cross-sectional survey adopted a 3-stage stratified random cluster sampling method to select representative samples of the general population aged 40 years or older in Dehui City of Jilin Province in Northeast China. In the first stage of sampling, 30 villages and 10 towns were randomly selected from 308 villages (rural) and 14 towns (urban) in Dehui City using the probability proportional to size (PPS) sampling method. In the second stage, 5 villagers' groups or communities were randomly sampled from both rural and urban strata using PPS. In the final stage, 1 adult resident aged 40 years or older was randomly selected from each household of the selected villagers' groups or communities. Respondents who were unwilling to participate in the survey or judged to be very frail were excluded. The study included 4052 subjects with the ratio of men to women being 0.67, which far exceeds the required sample size according to the 16.8% prevalence of diabetes in Chinese adults over 40 years of age [5].

### 2.2. Inclusion and Exclusion Criteria

Enrollment criteria for the target survey population:

1. Household registration location in Dehui City, Jilin Province, and age ≥ 40 years old (time as of December 31, 2015)
2. Permanent residents with an annual residence time of ≥6 months
3. Those who were able to sign an informed consent form, voluntarily participated in the survey of the project, and agreed to complete a face-to-face or telephone follow-up visit 1 year later

Exclusion criteria:

1. Household registration location other than Dehui City, Jilin Province, or age less than 40 years old (time as of December 31, 2015)
2. Residents with an annual residence time of <6 months
3. Those who are unwilling or unable to participate and cooperate in completing the survey of the project due to their physical health condition and other reasons

### 2.3. Data Collection

Face-to-face interviews or physical examinations were the methods of data collection. The data consisted of three parts: general information (e.g., sociodemographic characteristics), anthropometric information (e.g., WC, height, and weight), and blood biochemical information (e.g., blood glucose and blood lipid levels). All participants' information was collected according to the same questionnaire guidelines. Additionally, the researchers received uniform training prior to data collection.

### 2.4. Ethics Approval

This study was approved by the human ethics and research ethics committee of the First Hospital of Jilin University (Approval No: 2015-R-250), and written informed consent was obtained from all of the participants.

### 2.5. Measurements

For measuring the weight, the subjects were asked to wear lightweight clothes without shoes in the early morning on an empty stomach, and an electronic scale (OMRON HNH-219) was used. The electronic scale had an associated height rod that was used to measure participants' height. While standing on the scale without shoes, participants were asked to stand upright on the electronic scale with their head, hips, and heels close to the measuring rod. And participants could not stand on their toes or raise their heads, and they were allowed to look straight ahead. An investigator then recorded the intersection of the highest point of the participant's head with the vertical line of the measuring rod as the height reading. Height is measured in the range of 70-200 cm, and weight is measured in the range of 5-200 kg. The measurement accuracy of height and weight was required to be 0.1 cm and 0.1 kg, respectively. When measuring the WC, the subject was asked to breathe normally, wear thin clothes, and the measuring tape was placed about 0.5-1.0 cm above the navel. For measuring the hips, the position of the greatest circumference at the buttocks was used. The measurement accuracy of WC and hips was required to be 0.1 cm. The subjects were asked to rest for 20 minutes before the systolic and diastolic blood pressures were measured with an electronic sphygmomanometer (OMRON HEM-7200). Blood glucose was measured by taking a blood sample at least eight hours after fasting. The blood samples were collected from the subjects in the morning after an overnight fast (at least 8 hours) and transported to the same laboratory (Changchun Kingmed Center for Clinical Laboratory Co., Ltd.) under refrigeration and then stored at -20°C. The laboratory finished the blood examinations within 8 hours after receiving the samples and provided daily quality control charts.

### 2.6. Definitions

Diabetes mellitus was defined as the use of insulin and/or oral hypoglycemic medications or a self-reported history of diabetes or FBG ≥ 7.0 mmol/L in the field survey [25]. If the subjects had a history of diabetes, they were required to provide detailed medical records, including the patient's medical history, family history, blood sugar control, and medication. On the basis of the smoking status, the subjects were divided into three groups: current smoker, previous smokers, and never smokers. Current smokers referred to participants who revealed the use of any type of tobacco product during the interview. Respondents who had quit smoking for more than three months were defined as former smokers. Never smokers were those who had never smoked or smoked less than 100 cigarettes in their lifetime [26]. A subject who consumed more than 42 g of pure alcohol per day or more than 98 g of pure alcohol per week was defined as a drinker [27]. In this study, a questionnaire survey was used to determine the physical activity levels. The questions included were as follows: How many times per week was physical activity undertaken (including industrial and agricultural labor)? How long did it last? If the frequency of weekly physical exercise (including industrial and agricultural labor) was less than three times, and the duration of each episode did not exceed 30 min, it was defined as irregular exercise.

### 2.7. Statistical Analysis

Data were described on the basis of the distribution characteristics. The chi-square test was used to compare the difference in diabetes prevalence between different groups. The values of BMI, WC, WHR, and WHtR were divided into quartiles (Q1: <25%; Q2: ~25%; Q3: ~50%; and Q4: ~75%). The median of each quartile was used for a linear trend test. The receiver operating characteristic (ROC) curve was used to compare the area under the curve (AUC) between the four anthropometric indices. The comparison of two ROC curves was performed by adopting the technique of Delong et al. [28]. Multivariate logistic regression analysis was used to determine the association between diabetes and body fat measurement indices. Three models adjusted with different covariates were used for sensitivity analysis. In Model 1, no covariates were adjusted. In Model 2, covariates were adjusted including age, education, and family history of diabetes. In Model 3, covariates were adjusted as much as possible, containing age, sex, area, education, smoking, drinking, family history of diabetes, and regular exercise. Through sensitivity analysis, our goal was to obtain the upper and lower limits of the odds ratio (OR) value. In Model 3, the final results included age, sex, region, education, smoking, drinking, family history of diabetes, and regular exercise. Since there were too many questions related to diet and psychological state in the survey, which could reduce the response rate of the subjects, we did not consider diet and psychological factors during this study. The multivariate logistic regression model was used to calculate the standard deviation and OR for each anthropometric variable. All tests were two-tailed, and *p* < 0.05 was considered statistically significant. All calculations were performed using SPSS 22.0 (IBM Corp., Armonk, NY, USA) or MedCalc (MedCalc, Mariakerke, Belgium).

## 3. Results

A total of 4052 participants over 39 years of age were included in this study. The mean age of the participants was 54.85 ± 9.30 years. The prevalence of diabetes was 9.8% (9.5% among men and 10.0% among women), and all patients were historical cases. No patients were newly diagnosed with diabetes during the study. Prevalence of diabetes varied according to the sex, age, region, and other characteristics of the participants (Table 1). Moreover, the prevalence of diabetes increased with age and was much higher in participants with a family history of diabetes than in those without a family history (20.1% vs. 7.7%, *p* < 0.001). Besides, the prevalence of diabetes in individuals with a college education or above (4.8%) was significantly lower than in other education groups (*p* = 0.004), including primary school and below (11.0%), junior middle school (9.7%), and senior middle school (10.6%).

Table 2 shows the OR and its 95% confidence intervals (CI) for the diabetes prevalence rate, according to quartiles of BMI, WC, WHR, and waist-to-height ratio. For all the four body fat-measuring indices including BMI (adjusted OR: 3.300, 95% CI: 2.370, 4.595), WC (adjusted OR: 5.131, 95% CI: 3.433, 7.669), WHR (adjusted OR: 3.327, 95% CI: 2.386, 4.638), and WHtR (adjusted OR: 5.959, 95% CI: 3.922, 9.054), patients in the highest quartile were more likely to have diabetes than those in the lowest quartile. In addition, the highest quartiles of WC and WHtR were 1.8 times more likely to have diabetes than the highest quartiles of BMI and the waist-hip ratio. The linear trend test demonstrated that the risk of diabetes increased with increasing BMI, WC, WHR, and WHtR (*p* < 0.001) after adjustment for age, sex, region, education, smoking, drinking, family history of diabetes, and regular exercise. Similarly, the risk of developing diabetes per standard deviation (SD) increased 1.530 times (95% CI: 1.379, 1.698) with BMI, 1.787 times (95% CI: 1.591, 2.007) with WC, 1.575 times (95% CI: 1.397, 1.775) with WHR, and 1.786 times (95% CI: 1.590, 2.006) with waist-to-height ratio.

**Figure 1:**
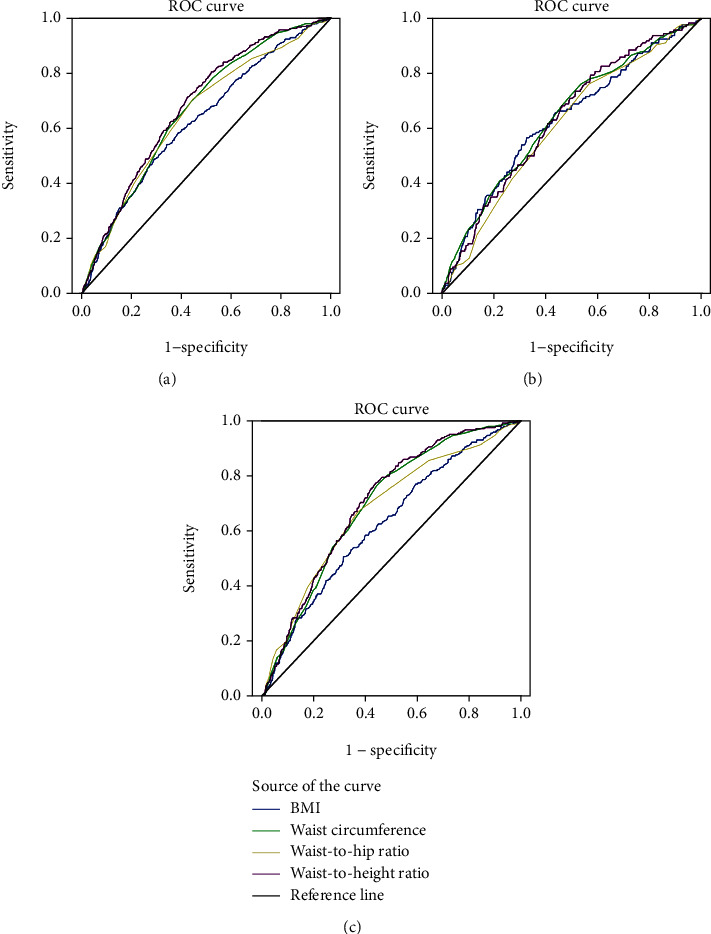
ROC curves of the body fat-measuring indices for diabetes (a). ROC curves of the body fat-measuring indices for diabetes in males (b). ROC curves of the body fat-measuring indices for diabetes in females (c).

Table 3 exhibits the AUC values and the optimum cut-off points of the body fat-measuring indices for diabetes. AUC values of WHtR, WC, WHR, and BMI for diabetes were 0.683 (95% CI: 0.657-0.709), 0.669 (95% CI: 0.643-0.695), 0.654 (95% CI: 0.626-0.682), and 0.629 (95% CI: 0.600-0.658), respectively (Table 3 and Figure 1(a)). When the optimum cut-off point of WHtR was 0.5337, the corresponding sensitivity was 71.11% and the specificity was 57.96%. In the case of WC, when the optimum cut-off point was 86.00, the corresponding sensitivity and specificity were 69.85% and 56.10%, respectively. When the optimum cut-off point of WHR was 0.89, the corresponding sensitivity and specificity were 70.35% and 55.83%, respectively. When the optimum cut-off point of the BMI ratio was 25.51, the corresponding sensitivity was 54.02% and specificity was 66.23% (Table 3). Compared to other indices in women, the AUC values of WHtR and WC were 0.710 (95% CI: 0.679-0.741) and 0.701 (95% CI: 0.670-0.732), respectively (Figure 1(c)). Furthermore, Youden's index for WHtR, WC, and WHR in women was 0.3434, 0.3301, and 0.3094, respectively. The results of the pairwise comparison of ROC curves are shown in Supplementary Tables 1-3.

## 4. Discussion

Since it has been reported that there is a significant increase in the incidence and prevalence of diabetes at the age of 40 years and above, our study included participants aged 40 years and above [5]. Further, our study confirmed that the risk factors associated with diabetes are obesity, age, family history of diabetes, and education. Since the prevalence of diabetes increases with age, the increase in the aging population of China is an important factor leading to the increase in diabetes prevalence [2] and people aged 40 years and above should be the main target group for diabetes prevention and screening in Jilin province.

This study revealed that BMI, WC, WHtR, and WHR are all related to the risk of diabetes. Similar to other studies, all four indicators play an important role in different populations [19–21]. After adjusting for multiple covariates, as the four indicators increase, the prevalence of diabetes increases. Among them, WC and WHtR have a stronger correlation with the prevalence of diabetes. The possible explanation is that WC and WHtR can better reflect the accumulation of abdominal fat or ectopic fat in diabetes [29]. Abdominal obesity is a more significant risk factor for metabolic diseases than general obesity indicators [17]. The adipose tissue-secreted factors may impair glucose tolerance and cause chronic inflammation in the adipose tissue as well as insulin resistance and damage to the pancreatic *β* cells, which may intensify the development of diabetes [30]. The risk factors for diabetes are age, sex, and genetic history, which are the broad causes of abdominal fat accumulation [31]. Since BMI cannot distinguish between fat and lean mass, WC is more reflective of visceral obesity than BMI [32]. Consistent with other research results [8, 11, 21, 33], as phenotypic markers of total fat and regional obesity [34], WC and WHtR can identify individuals with lower body weight but increased ectopic fat accumulation in order to prevent the development of diabetes [35, 36]. Since hyperglycemia is a chronic gradually developing process, it is usually not enough in the early stages to manifest any noticeable classic diabetes symptoms; however, there may be an increase in the proportion of body fat [3]. Therefore, using BMI combined with WHtR, WC, and WHR can improve the risk phenotyping of diabetes and screen prediabetic patients.

Another important finding of the study is that, compared to men, the value of WC and WHtR in diagnosing diabetes is higher than other indicators in women aged 40 years and above. A possible explanation is the sex differences in visceral fat deposition and regional fat tissue distribution [37]. Due to the changes in the body fat distribution during the menopausal state, the abdominal fat deposition in postmenopausal women becomes more obvious [38]. Therefore, the relationship between female visceral adipose tissue and risk factors for diabetes is stronger. These results are consistent with the results of cross-ethnic studies and case-control studies [14, 39], which have reported that high WC and WHtR are important risk factors for diabetes among women. A study has reported that 12.2% of normal weight people have a higher WC, and 13.3% of women have a higher WHtR, which indicates that some people who are not obese may have abdominal fat distribution [40]. It can be hypothesized that the optimum cut-off points of 0.5337 for WHtR and 84.00 cm for WC may be utilized for diabetes screening in women aged 40 years and above in Jilin Province.

Due to different demographic characteristics, the WHtR of 0.5 is used as the optimum cut-off for predicting diabetes globally [41], and based on our study findings, a WHtR of 0.5337 is recommended as the optimum cut-off for predicting diabetes in Jilin Province, China. According to a meta-analysis [42], WC ≥ 88 cm is a predictive indicator of diabetes in women irrespective of race and age. In light of the study results and considering the population characteristics of Jilin Province, a WC ≥ 84 cm is recommended as the optimum cut-off for predicting diabetes. Indeed, the determination of the critical value needs further research and exploration.

This study has some limitations. Firstly, the participants were recruited from only Jilin Province. The prevalence and incidence rates of diabetes in Jilin Province are different from other regions of China, and the conclusion cannot be generalized to other regions. Secondly, this was a cross-sectional study. Consequently, it cannot be used to establish temporal and causal relationships. However, it can provide clues about risk factors for further research. Thirdly, the dietary intake and work-related physical activity were not evaluated in multivariate logistic regression analysis, and therefore, an association between these factors and the prevalence of diabetes cannot be established. Finally, the nature of the self-reported data and cross-sectional data may lead to recalls and reporting deviations, which may affect the accuracy of the results. Nevertheless, the advantages of this research study lie in the representative sample survey and high response rate.

## 5. Conclusions

The WC and WHtR were more closely related to diabetes than BMI and WHR among study participants ≥ 40 years of age, especially in females.

## Figures and Tables

**Table 1:** Characteristics of the included participants aged 40 years or older (*n* = 4052).

Characteristics	Diabetes		*χ* ^2^	*p*
	Yes	No		
*Sex*			0.293	0.588
Male	154 (9.5)	1465 (90.5)		
Female	244 (10.0)	2189 (90.0)		
*Age*			46.527	<0.001^∗^
40~	77 (5.6)	1299 (94.4)		
50~	150 (10.9)	1222 (89.1)		
60~	128 (12.7)	881 (87.3)		
70~	43 (14.6)	252 (85.4)		
*Area*			1.829	0.169
Urban	190 (9.2)	1877 (90.8)		
Rural	208 (10.5)	1777 (89.5)		
*Education*			13.187	0.004^∗^
Primary school and below	159 (11.0)	1287 (89.0)		
Junior middle school	164 (9.7)	1532 (90.3)		
Senior middle school	57 (10.6)	480 (89.4)		
College and above	18 (4.8)	355 (95.2)		
*Smoking*			0.768	0.381
Yes	127 (9.2)	1246 (90.8)		
No	271 (10.1)	2408 (89.9)		
*Drinking*			3.433	0.064
Yes	90 (8.4)	984 (91.6)		
No	308 (10.3)	2670 (89.7)		
*Family history of diabetes*			100.586	<0.001^∗^
Yes	141 (20.1)	562 (79.9)		
No	257 (7.7)	3092 (92.3)		
*Regular exercise*			2.477	0.116
Yes	297 (9.4)	2853 (90.6)		
No	101 (11.2)	801 (88.8)		

**Table 2:** Odds ratio (95% confidence intervals) for diabetes, according to the quartiles of BMI, WC, WHR, and WHtR.

Body fat-measuring indices	Quartiles of body fat-measuring indices				*p* for trend	Per SD increase
	Q1	Q2	Q3	Q4		
*BMI (kg/m^2^)*	<22.19	22.19-24.22	24.23-26.63	≥26.64	—	—
No. of events	1006 (24.8)	1019 (25.1)	1011 (25.0)	1016 (25.1)	—	—
Model 1^∗^	1	1.604 (1.127, 2.283)	1.892 (1.341, 2.670)	3.320 (2.406, 4.580)	<0.001^∗^	1.543 (1.395, 1.705)
Model 2^†^	1	1.655 (1.156, 2.370)	1.902 (1.339, 2.702)	3.248 (2.337, 4.514)	<0.001^∗^	1.525 (1.375, 1.691)
Model 3^‡^	1	1.665 (1.162, 2.386)	1.932 (1.358, 2.747)	3.300 (2.370, 4.595)	<0.001^∗^	1.530 (1.379, 1.698)
*WC (cm)*	<80.3	80.3-84.2	84.3-90.9	≥91.0	—	—
No. of events	978 (24.1)	859 (21.2)	1141 (28.2)	1074 (26.5)	—	—
Model 1^∗^	1	2.062 (1.322, 3.215)	4.067 (2.741, 6.036)	5.637 (3.824, 8.311)	<0.001^∗^	1.826 (1.639, 2.034)
Model 2^†^	1	1.972 (1.258, 3.089)	3.500 (2.342, 5.229)	4.754 (3.200, 7.061)	<0.001^∗^	1.725 (1.541, 1.930)
Model 3^‡^	1	2.003 (1.277, 3.141)	3.693 (2.465, 5.533)	5.131 (3.433, 7.669)	<0.001^∗^	1.787 (1.591, 2.007)
*WHR*	<0.8800	0.8800-0.8946	0.8947-0.9037	≥0.9038	—	—
No. of events	1009 (24.8)	1000 (24.7)	963 (23.8)	1080 (26.7)	—	—
Model 1^∗^	1	1.030 (0.695, 1.526)	2.422 (1.721, 3.408)	3.705 (2.686, 5.111)	<0.001^∗^	1.679 (1.493, 1.886)
Model 2^†^	1	1.064 (0.712, 1.590)	2.192 (1.543, 3.115)	3.190 (2.296, 4.433)	<0.001^∗^	1.580 (1.402, 1.781)
Model 3^‡^	1	1.050 (0.695, 1.586)	2.285 (1.591, 3.281)	3.327 (2.386, 4.638)	<0.001^∗^	1.575 (1.397, 1.775)
*WHtR*	<0.4880	0.4880-0.5272	0.5273-0.5651	≥0.5652	—	—
No. of events	1011 (25.0)	1010 (24.9)	1017 (25.1)	1014 (25.0)	—	—
Model 1^∗^	1	2.522 (1.621, 3.923)	4.530 (2.990, 6.864)	7.259 (4.851, 10.862)	<0.001^∗^	1.875 (1.686, 2.085)
Model 2^†^	1	2.335 (1.493, 3.651)	3.968 (2.599, 6.056)	5.965 (3.930, 9.053)	<0.001^∗^	1.786 (1.592, 2.003)
Model 3^‡^	1	2.348 (1.500, 3.675)	4.006 (2.622, 6.121)	5.959 (3.922, 9.054)	<0.001^∗^	1.786 (1.590, 2.006)

^∗^Unadjusted. ^†^Adjusted for age, education, and family history of diabetes. ^‡^Adjusted for age, sex, area, education, smoking, drinking, family history of diabetes, and regular exercise.

**Table 3:** The area under the curve and the optimum cut-off points of the body fat-measuring indices for diabetes.

Test variables	Area under the curve	95% confidence interval		Optimum cut-off point	Sensitivity (%)	Specificity (%)	Youden's index
		Lower bound	Upper bound				
*Total*							
BMI	0.629	0.600	0.658	25.51	54.02	66.23	0.2025
WC	0.669	0.643	0.695	86.00	69.85	56.10	0.2595
WHR	0.654	0.626	0.682	0.89	70.35	55.83	0.2618
WHtR	0.683	0.657	0.709	0.5337	71.11	57.96	0.2907
*Male*							
BMI	0.629	0.581	0.676	25.99	56.49	67.37	0.2387
WC	0.639	0.593	0.685	87.00	75.97	46.48	0.2246
WHR	0.609	0.565	0.654	0.89	75.97	43.41	0.1939
WHtR	0.634	0.590	0.679	0.5137	79.22	42.80	0.2202
*Female*							
BMI	0.633	0.596	0.669	25.49	50.41	69.67	0.2008
WC	0.701	0.670	0.732	84.00	76.23	56.78	0.3301
WHR	0.684	0.648	0.719	0.89	66.80	64.14	0.3094
WHtR	0.710	0.679	0.741	0.5337	77.05	57.29	0.3434

## Data Availability

The data sets generated for this study are available on request to the corresponding author.

## Supplementary Materials

Supplementary Materials Table S1: pairwise comparison of ROC curves of body fat-measuring indices for diabetes (Z, P). Table S2: pairwise comparison of ROC curves of body fat-measuring indices for diabetes in males (Z, P). Table S3: pairwise comparison of ROC curves of body fat-measuring indices for diabetes in female (Z, P).
