# Supplementary material for: Strong Association of Waist Circumference (WC), Body Mass Index (BMI), Waist-to-Height Ratio (WHtR), and Waist-to-Hip Ratio (WHR) with Diabetes: A Population-Based Cross-Sectional Study in Jilin Province, China
Source: J Diabetes Res. 2021 May 14;2021:8812431. doi: 10.1155/2021/8812431 (PMC8147550; doi:10.1155/2021/8812431)
Supplement: Supplementary Materials — Table S1: pairwise comparison of ROC curves of body fat-measuring indices for diabetes (Z, P). Table S2: pairwise comparison of ROC curves of body fat-measuring indices for diabetes in males (Z, P). Table S3: pairwise comparison of ROC curves of body fat-measuring indices for diabetes in female (Z, P). [file 8812431.f1.pdf]

**Table S1. Pairwise comparison of ROC curves of body fat measuring indices for diabetes. (Z, P)**

| Indicators | BMI | WC | WHR | WHtR |
|---|---|---|---|---|
| **BMI** | -- | 3.732, <0.001 | 1.621,0.105 | 4.620, <0.001 |
| **WC** | 3.732, <0.001 | -- | 1.607,0.108 | 1.946,0.052 |
| **WHR** | 1.621,0.105 | 1.607,0.108 | -- | 2.692,0.007 |
| **WHtR** | 4.620, <0.001 | 1.946,0.052 | 2.692,0.007 | -- |

**Table S2. Pairwise comparison of ROC curves of body fat measuring indices for diabetes in male. (Z, P)**

| Indicators | BMI | WC | WHR | WHtR |
|---|---|---|---|---|
| **BMI** | -- | 0.632,0.528 | 0.899,0.369 | 0.337,0.736 |
| **WC** | 0.632,0.528 | -- | 2.140,0.032 | 0.525,0.599 |
| **WHR** | 0.899,0.369 | 2.140,0.032 | -- | 1.633,0.103 |
| **WHtR** | 0.337,0.736 | 0.525,0.599 | 1.633,0.103 | -- |

**Table S3. Pairwise comparison of ROC curves of body fat measuring indices for diabetes in female. (Z, P)**

| Indicators | BMI | WC | WHR | WHtR |
|---|---|---|---|---|
| **BMI** | -- | 4.945, <0.001 | 2.496,0.013 | 5.418, <0.001 |
| **WC** | 4.945, <0.001 | -- | 1.351,0.177 | 1.361,0.173 |
| **WHR** | 2.496,0.013 | 1.351,0.177 | -- | 1.863,0.063 |
| **WHtR** | 5.418, <0.001 | 1.361,0.173 | 1.863,0.063 | -- |
